# Non-destructive wood identification using X-ray µCT scanning: which resolution do we need?

**DOI:** 10.1186/s13007-024-01216-0

**Published:** 2024-06-24

**Authors:** Sofie Dierickx, Siska Genbrugge, Hans Beeckman, Wannes Hubau, Pierre Kibleur, Jan Van den Bulcke

**Affiliations:** 1 Cultural Anthropology and History Department, Royal Museum for Central Africa, Leuvensesteenweg 7, 3080 Tervuren, Belgium; 2 UGent-Woodlab-Laboratory of Wood technology, Department of Environment, Faculty of Bioscience Engineering, Ghent University, Proeftuinstraat 86/N12, 9000 Ghent, Belgium; 3 Wood Biology Department, Royal Museum for Central Africa, Leuvensesteenweg 7, 3080 Tervuren, Belgium; 4 Radiation Physics Research Group, Department Physics and Astronomy, Ghent University, Proeftuinstraat 86/N12, 9000 Ghent, Belgium; 5 UGCT, Ghent University, Proeftuinstraat 86/N12, 9000 Ghent, Belgium

**Keywords:** X-ray µCT-scanning, Tropical wood species, Wood identification

## Abstract

**Background:**

Taxonomic identification of wood specimens provides vital information for a wide variety of academic (e.g. paleoecology, cultural heritage studies) and commercial (e.g. wood trade) purposes. It is generally accomplished through the observation of key anatomical features. Classic methodologies mostly require destructive sub-sampling, which is not always acceptable. X-ray computed micro-tomography (µCT) is a promising non-destructive alternative since it allows a detailed non-invasive visualization of the internal wood structure. There is, however, no standardized approach that determines the required resolution for proper wood identification using X-ray µCT. Here we compared X-ray µCT scans of 17 African wood species at four resolutions (1 µm, 3 µm, 8 µm and 15 µm). The species were selected from the Xylarium of the Royal Museum for Central Africa, Belgium, and represent a wide variety of wood-anatomical features.

**Results:**

For each resolution, we determined which standardized anatomical features can be distinguished or measured, using the anatomical descriptions and microscopic photographs on the Inside Wood Online Database as a reference. We show that small-scale features (e.g. pits and fibres) can be best distinguished at high resolution (especially 1 µm voxel size). In contrast, large-scale features (e.g. vessel porosity or arrangement) can be best observed at low resolution due to a larger field of view. Intermediate resolutions are optimal (especially 3 µm voxel size), allowing recognition of most small- and large-scale features. While the potential for wood identification is thus highest at 3 µm, the scans at 1 µm and 8 µm were successful in more than half of the studied cases, and even the 15 µm resolution showed a high potential for 40% of the samples.

**Conclusions:**

The results show the potential of X-ray µCT for non-destructive wood identification. Each of the four studied resolutions proved to contain information on the anatomical features and has the potential to lead to an identification. The dataset of 17 scanned species is made available online and serves as the first step towards a reference database of scanned wood species, facilitating and encouraging more systematic use of X-ray µCT for the identification of wood species.

## Background

Verifying which wood species are present in woody products is of great interest to many research fields, both academic and commercial. Wood traded internationally may need to be identified to enforce international restrictions for endangered species such as those listed in the Convention on International Trade in Endangered Species of Wild Fauna and Flora (CITES) [1]. Archaeological or art objects containing wooden elements may also be legally required to provide a wood species identification before allowing international travel. In addition, the identification of wood species can offer important information about the context of the wooden sample [2, 3]. In the field of paleoecology, the identification of wood or charcoal samples can offer insight into the evolution of forests throughout history [4–6]. In the field of archaeology and art history, the identification of materials can add invaluable information about the object’s history, use and best preservation [7, 8].

To date, the most common method for identifying wood species is through destructive sampling of a small piece of wood and studying the wood anatomical features microscopically. In many fields, taking a sample for destructive analysis does not pose a problem. Yet in other cases (e.g. cultural objects), even a small sample constitutes a large gap in the original material and an irreversible change of the integrity of the object.

To answer the need for non-destructive wood identification, several minimally- or non-destructive tools for anatomical analysis are being investigated and developed. Chemical profiling, using the extraction of mass spectral ions, is increasingly implemented for wood identification [9–11]. DNA analysis of wood species, while promising, depends on databases still in early development [12, 13]. More recently, micro-magnetic resonance imaging has been explored to study wood anatomy [14].

However, X-ray tomography is by far the most developed non-destructive analysis technique. This tool can visualize the same anatomical features that standard microscopic analysis relies on. At the first implementation of X-ray CT in the medical field, the quality of the scans was too low to visualize the wood for any anatomical information. As technology advanced, and subsequently the quality of the scans improved, X-ray CT was adopted in studies of plant tissue [15–18]. From 2009 onwards, promising experiments and case studies have been published, proving the viability of the technique for descriptive and quantitative wood identification [19, 20].

When considering X-ray µCT for identification of a wooden artefact, the question remains which resolution will be sufficient to visualize the wood anatomical features. The dimensions of the scanned sample and the technical limits of the scanning system determine the resolution and hence successful identification. The degree of detail of the scans determines whether the anatomical features can be distinguished, and whether quantitative features can be measured or counted. As such, while previous case studies have shown that X-ray CT scanning can be successful [19, 21–24], each new object considered for scanning must undergo an experimental approach.

This paper compares the success of wood anatomical description among four different resolutions between from 1 to 15 µm. In order to attain a controlled range of resolutions that are unaffected by object-specific constraints such as dimensions or shape, small cubes were cut for this experiment. These samples were collected from the Tervuren Xylarium (Royal Museum for Central Africa, RMCA) and comprise 17 African species that are heterogeneous in terms of anatomical structures (Table 1). The baseline for the evaluation of the scans were the descriptions and microscopic photographs on the Inside Wood online database [25]. The resulting overview describes which features could be observed at the four resolutions. Finally, following this list of observable features, an assessment is made of the possibilities of a successful species identification at each resolution.

**Table 1:** list of 17 African wood species scanned for this study, ranked according to the number of objects identified in the Congolese heritage collection of the RMCA, with examples of the types of objects made of the wood species

17 wood species scanned	Objects identified	Types of African artefacts in collection most manufactured from this wood species
RUBIACEAE *Crossopteryx febrifuga* Afzel. Ex G. Don	844	Sculptures (323), power objects (171), cups (84)
EUPHORBIACEAE *Ricinodendron heudelotii* (Baill.) Pierre ex Heckel	265	Drums (139), masks (115)
APOCYNACEAE *Alstonia congensis* Engl.	223	Masks (112), drums (43), sculptures (31)
BURSERACEAE *Canarium schweinfurthii* Engl.	84	Power objects (57), sculptures (27)
RUBIACEAE *Nauclea pobeguinii* (Pobeg.) Merr.	71	Sculptures (58)
VERBENACEAE *Vitex madiensis* Oliv.	69	Sculptures (36), power objects (17)
BORAGINACEAE *Cordia millenii* Baker	60	Drums (53)
MORACEAE *Milicia excelsa* (Welw.) C.C. Berg	49	Drums (13), sculptures (13), power objects (11)
RUBIACEAE *Nauclea latifolia* Sm.	35	Sculptures (23), power objects (10)
BIGNONIACEAE *Markhamia tomentosa* (Benth.) K. Schum. Ex Engl.	33	Drums (29)
FABACEAE *Albizia zygia* (DC.) J.F. Macbr.	29	Head rests (16), drums (5)
FABACEAE *Pterocarpus tinctorius* Welw.	27	Power objects (7), staves (6), xylophones (4)
FABACEAE *Pterocarpus angolensis* DC.	21	Xylophones (9), drums (5)
APOCYNACEAE *Funtumia Africana* (Benth.) Stapf	18	Power objects (8), sculptures (5)
MORACEAE *Ficus mucuso* Welw. ex Fical	5	Drum (2)
VERBENACEAE *Vitex ferruginea* Schumach. & Thonn.	2	Sculptures (2)
OLACACEAE *Strombosiopsis tetranda* Engl.	0	/

## Methods

### Wood samples

The 17 wood species studied in this paper were selected according to their occurrence among the objects of the Congolese heritage collection of the RMCA (Table 1). In the past, 7% (3814 out of 55,000 objects) of the museum’s collection of wooden cultural objects was analysed and identified by the wood biology department of the RMCA. Based on this dataset of identifications, the most occurring wood species were selected (Table 1). Reference samples for each of these species were collected from the Tervuren Xylarium of the RMCA, which holds over 14,000 wood species from all over the world [26, 27]. To ensure correct identification, all 17 reference samples were taken from specimens in the Tervuren Xylarium that also have an herbarium sample in the Botanic Garden of Meise [28].

The selected species represent a large variety in anatomical structure, together containing 142 of the 163 features described on the IAWA list of anatomical features. This ensures that a wide range of wood anatomical features can be analysed at the four resolutions. To further complete the wood anatomical variety, a species with scalariform perforations *(Strombosiopsis tetrandra)* was added to the selection, despite its absence in the identified database. Every sample was prepared for scanning by subdividing it into small cubes, each tuned to the desired scan resolution: the smaller the sample, the closer it could be positioned to the scanner's X-ray source, resulting in a larger magnification of the internal wood structure. For the 1 µm scans cubes of 1 × 1 × 10 mm were cut; cubes of 5 × 5 × 5 mm were made for the 3 µm resolution scans, and for the 8 µm and 15 µm scans the cubes were cut to 1 × 1 × 1 cm.

### Nanowood X-ray µCT scanner

The wood samples were scanned at 4 different ‘resolutions’ -more correctly the approximate voxel pitch of the scans—hereafter referred to as resolution. The Nanowood X-ray µCT scanner was used to make the 68 scans (17 wood species at 4 resolutions). This scanner was custom-built at the UGent Centre for X-ray Tomography (www.ugct.ugent.be) and recently refurbished in collaboration with TESCAN-XRE (www.XRE.be, part of the TESCAN ORSAY HOLDINGS a.s.), a UGCT spin-off company. It is specifically designed to study materials made of wood or derived from wood. To visualize the various wood anatomical features on both larger and smaller length scales, Nanowood is equipped with two X-ray sources. The Hamamatsu transmission source has a 130 kV X-ray tube, a spot size down to 5 µm and a maximum power of 39 W. This source is most suited for larger samples, ranging from a few millimetres to several centimetres. The nanofocus transmission source can generate up to 100 kV, with a focal spot of 400 nm and a maximum power of 3 W, able to make sub-µCT scans [29]. To accommodate the varying X-ray energies emitted by both sources, Nanowood has two detectors with complementary spectral sensitivity. Table 2 shows the specifics for each resolution.

**Table 2:** Configuration and spectra used for the scans at each scanned resolution

Voxel size	Source	Detector	Tube voltage	Tube power	# Of projections	Exposure time
1 µm	Nanofocus transmission	Photonic detector	70 kV	4 W	2401	31 min
3 µm	Hamamatsu microfocus	Varian flatpanel	70 kV	7 W	2401	42 min
8 µm	Hamamatsu microfocus	Varian flatpanel	70 kV	7 W	2401	22 min
15 µm	Hamamatsu microfocus	Varian flatpanel	70 kV	7 W	2401	17 min

All scans in this study were reconstructed using the Octopus Reconstruction software [30]. The 1 µm scans were additionally phase filtered using the Paganin algorithm [31]. The reconstructed volumes were further processed using the open-source software ImageJ [32] for reslicing in the transverse, radial and tangential direction, as well as for quantitative measurements of anatomical features. VGStudioMAX (Volume Graphics, Germany) was used to generate 3D renderings [33].

### Inside wood

Inside Wood (insidewood.lib.ncsu.edu), the online reference database which contains wood anatomical descriptions of over 7.000 modern hardwood species [25], was used. This database is internationally recognised and uses the IAWA list of anatomical features to describe wood anatomy [34]: a total of 163 anatomical features is numbered and described, illustrated with microscopic images. Each wood species description is presented as a ‘string’ of numbers that are ‘present’ in the wood anatomy of the species. Features that are uncertain are marked with a ‘?’ and those with a ‘v’ are variable.

### Observation success and identification potential

The Inside Wood anatomical descriptions were used to evaluate which features can be observed on the µCT scans at each of the four resolutions. For each of the 68 scans (17 species × 4 resolutions), we started from the Inside Wood description of numbered features for each species. From that string, we then deleted those features that could not be distinguished or measured on the scan. For features that involve a measurement, we followed the recommendations of the IAWA committee: requiring a view of the entire measurable element, as well as a minimum number of elements to obtain an average value [34].

As such, for each of the scans, a shortened string remained, retaining only the features that could be confidently observed at that specific resolution. We then evaluated the observation success for each wood anatomical feature and for each resolution, expressed as a fraction: the number of samples with the feature observed on the scans versus the number of samples that have this feature according to Inside Wood.

For each of the 68 scans, we then entered the shortened strings of anatomical features in the Inside Wood search engine, resulting in a list of species. The lower the number of species, the higher the identification potential of the scan. We grouped the scans in three categories: high identification potential (≤ 20 species returned by Inside Wood), medium identification potential (> 20 and ≤ 40 species), low identification potential (> 40 species returned by Inside Wood).

## Results

### Observation success of wood anatomical features

**Fig. 1:**
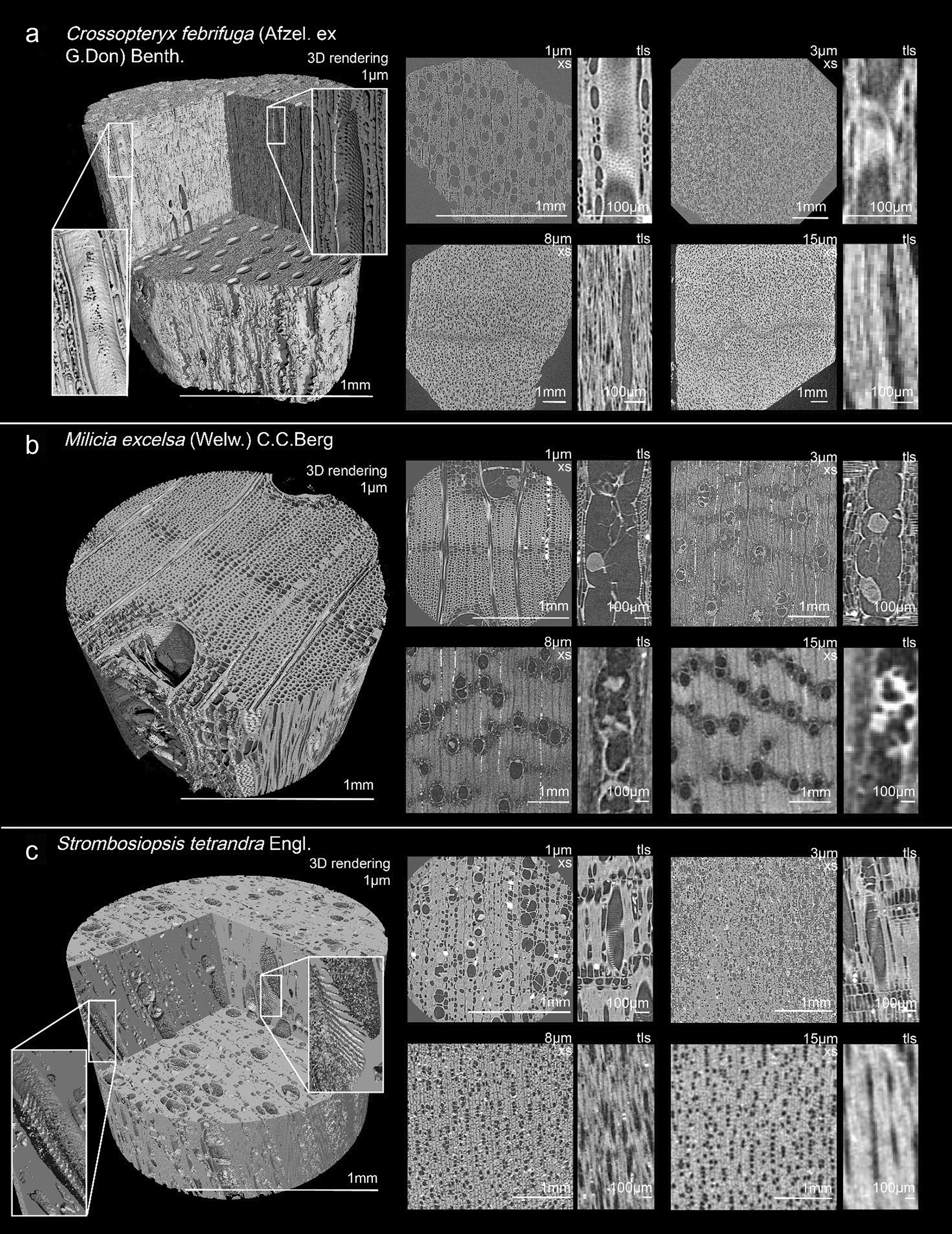
Illustrate observed features in the scanned samples. For each sample, a 3D rendering of the highest resolution (1 µm voxel size) is shown, as well as digital reslices of the transverse (XS), radial (RLS) or tangential (TLS) planes at different resolutions. **a**: *Crossopteryx febrifuga*, **b**: *Milicia excels*, **c**: *Strombosiopsis tetrandra*

**Fig. 2:**
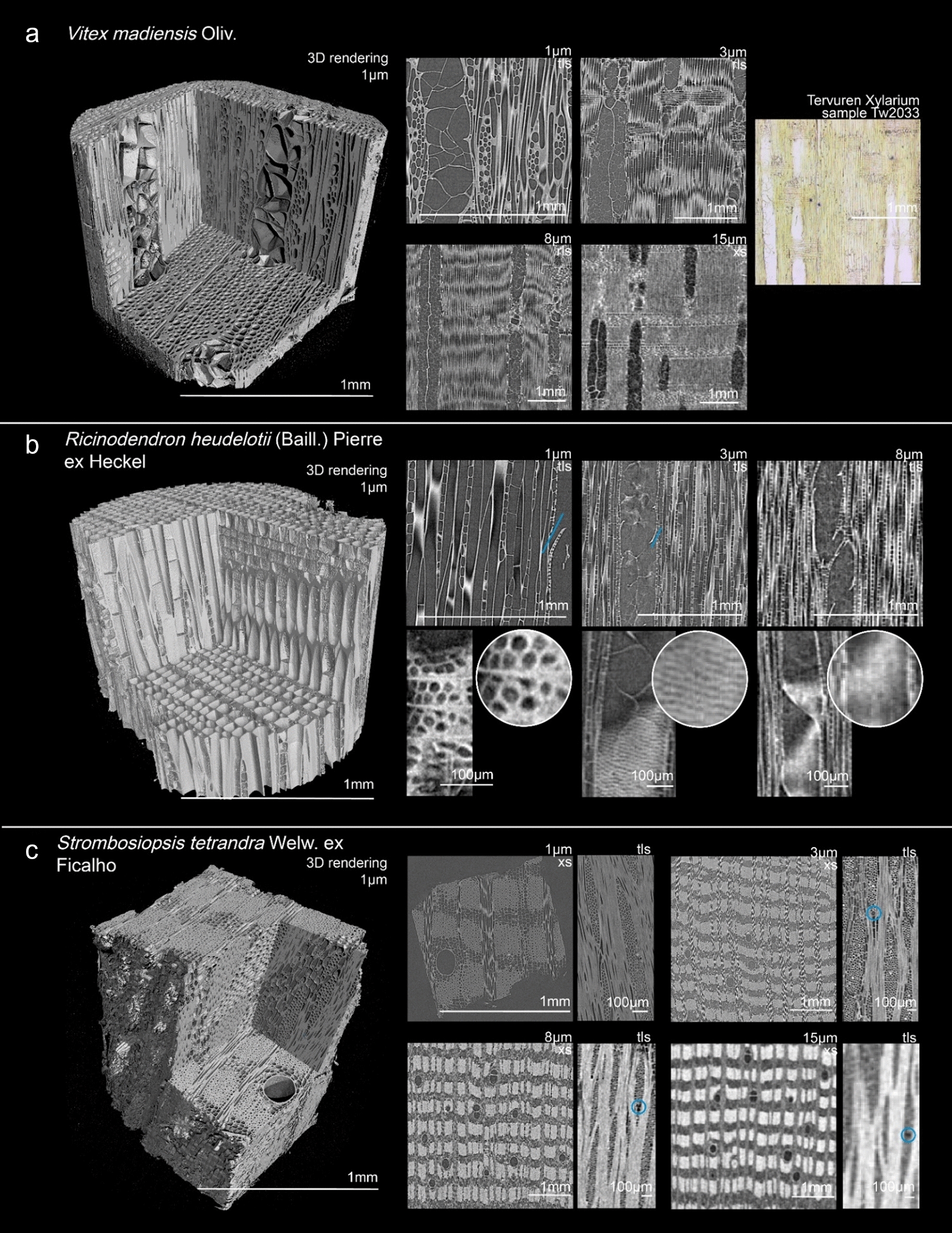
Illustrate observed features in the scanned samples. For each sample, a 3D rendering of the highest resolution (1 µm voxel size) is shown, as well as digital reslices of the transverse (XS), radial (RLS) or tangential (TLS) planes at different resolutions. **a**: *Vitex madiensis*, **b**: *Ricinodendron heudelotii*, **c**: *Ficus mucuso*

**Fig. 3:**
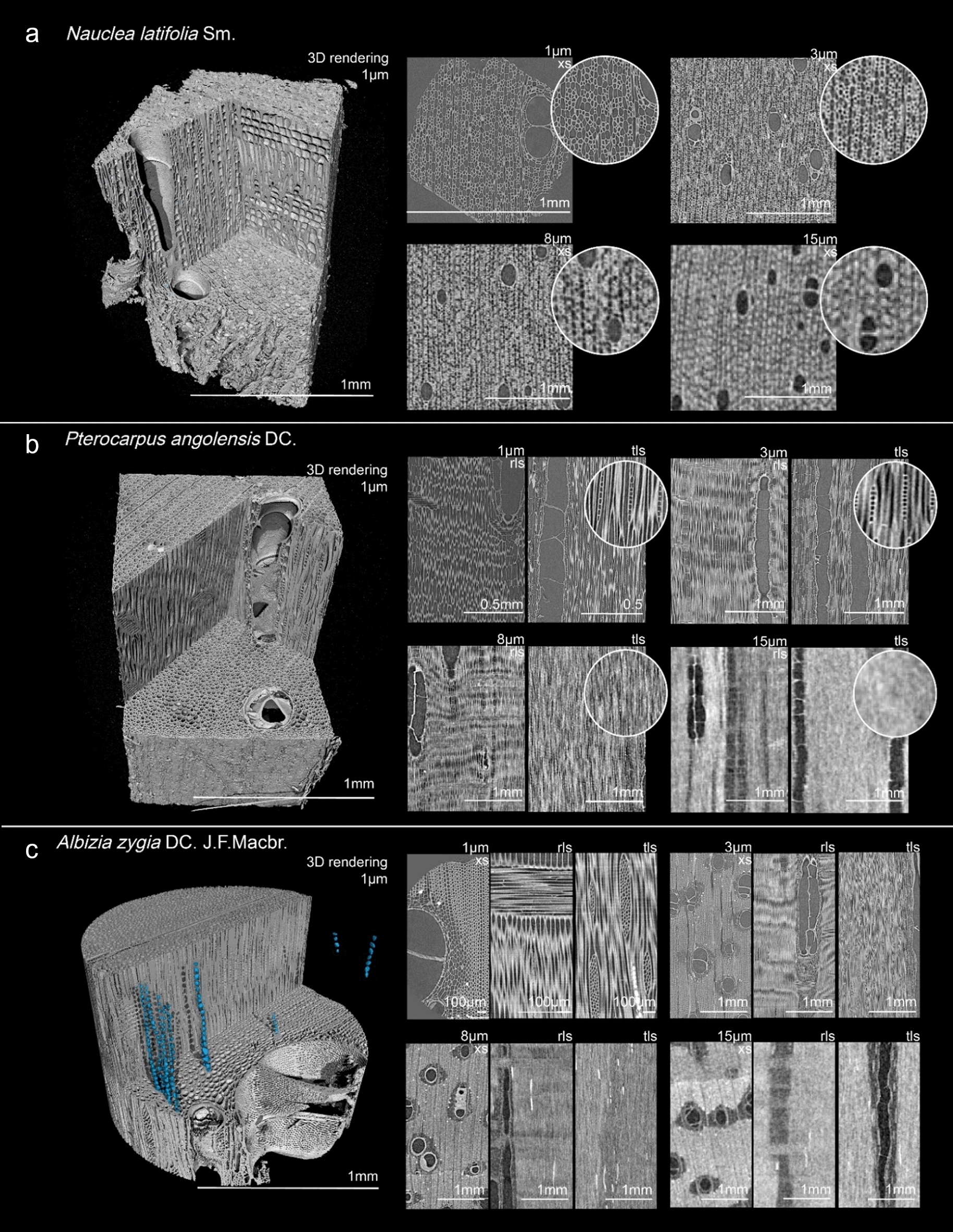
Illustrate observed features in the scanned samples. For each sample, a 3D rendering of the highest resolution (1 µm voxel size) is shown, as well as digital reslices of the transverse (XS), radial (RLS) or tangential (TLS) planes at different resolutions. **a***: Nauclea latifolia*, **b**: *Pterocarpus angolensis*, **c**: *Albizia zygia*

**Fig 4:**
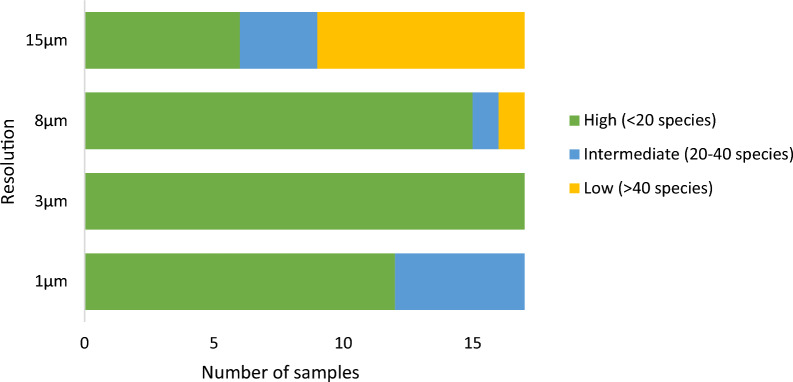
Identification potential per resolution. High identification potential is presented in green (≤ 20 species returned by Inside Wood), medium identification potential in blue (> 20 and ≤ 40 species), and low identification potential in orange (> 40 species returned by Inside Wood)

As indicated by the bottom row of Table 3, the highest observation success rate was recorded on the 3 µm resolution scans with 25 feature categories observed in 80% or more of the scans. On the 8 µm scans, 17 feature-categories scored a high observation success rate. At the highest resolution scanned, 1 µm, 14 feature categories have a high observation success rate, while at 15 µm only 6 categories have a high observation success rate. Figures 1a–c, 2a–c, 3a–c, 4 serve as illustrations. Each figure shows a 3D rendering of a scan, and the 2D transverse, radial, and tangential resliced scans at the four resolutions.

**Table 3:**
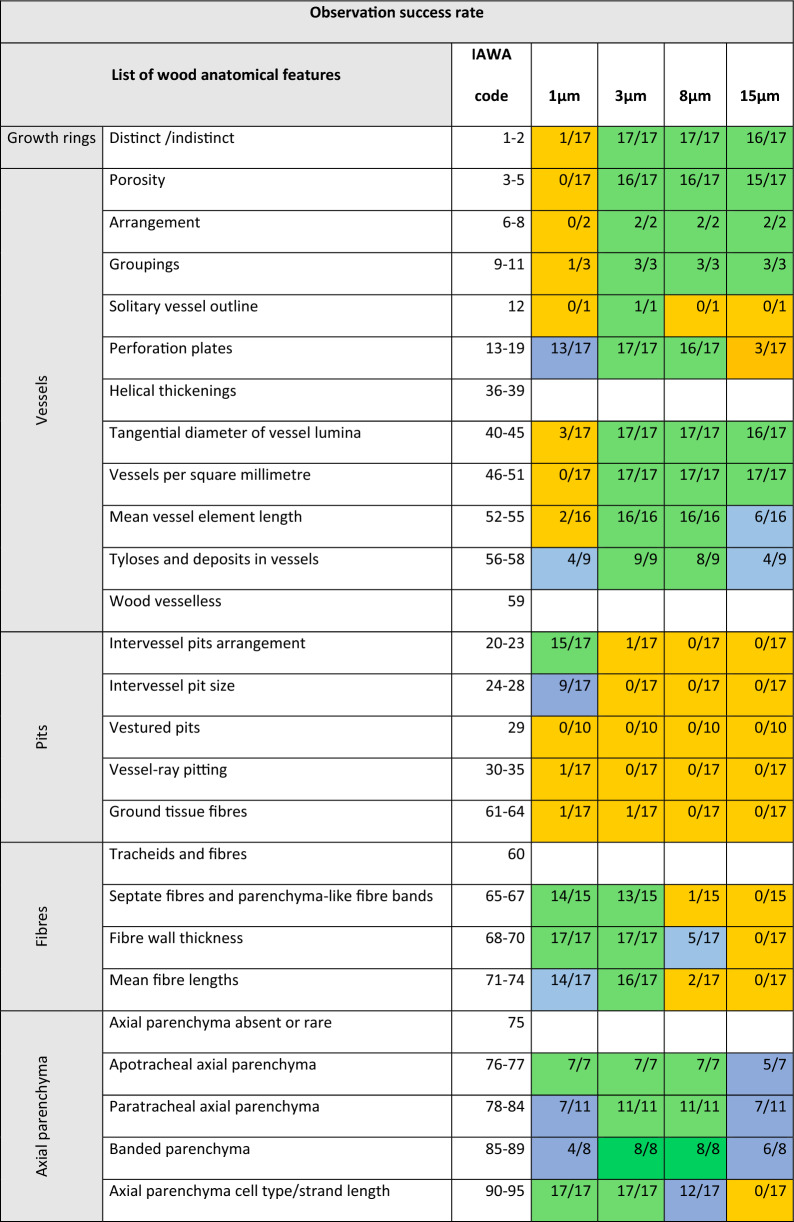

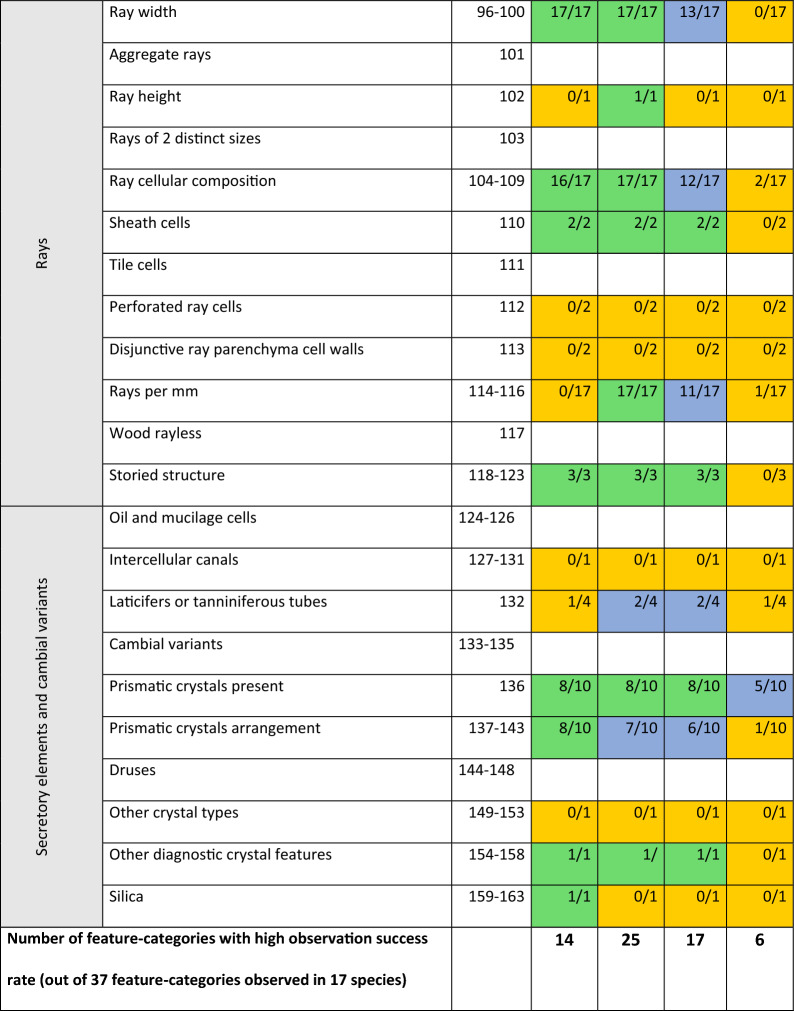
Overview of how often features from each anatomical category were recorded at the 4 resolutions

The colours indicate if the observation success rate was ≥ 80% (green), between 80 and 20% (blue) or < 20% (orange). Empty cells indicate features that are not present in any of the 17 scanned species

#### Growth ring boundaries

Growth ring boundaries can be distinguished (either as distinct or indistinct) in all scans at 3 µm, 8 µm and 15 µm. At the highest resolution, they can be distinguished in only 6% (1/17) of the scans. This can be explained by the small field of view offered by the scans at 1 µm voxel size—a little over 1 mm^2^—which lowers the probability of growth ring boundaries being captured in the scan.

Figure 1a shows the scanned sample of *Crossopteryx febrifuga*, which is described on Inside Wood as having indistinct growth ring boundaries. On the 1 µm and 3 µm scans there are indeed no structural changes visible in the wood structure on the cross-sections. On the 8 µm and 15 µm however, the density variations in the wood structure could be interpreted as a growth ring boundary. Figure 1b shows the scanned sample of *Milicia excelsa*, a species described on Inside Wood as having indistinct growth ring boundaries, but variably distinct (1v and 2p). Of all 17 scanned samples, *Milicia excelsa* is the only one with an observable growth ring boundary at the highest resolution (1 µm) scan. At the lower resolution scans, the growth ring boundaries can also be distinguished (indicated with a blue arrow).

#### Vessel elements

The observation success rate of vessel element features (Table 3) is generally low for the highest resolution (1 µm), but much higher for the scans at 3 µm, 8 µm and even 15 µm voxel size. This is again explained by the small size of the sample scanned at 1 µm, resulting in a limited field of view. For many of the scanned samples, this meant that the highest resolution scan has not enough vessel elements to draw any conclusions about arrangement, grouping and size. Of the 17 studied species only 3 presented vessel elements small and numerous enough to describe them on the 1 µm scans: *Vitex ferruginea, Crossopteryx febrifuga* (Fig. 1a) and *Strombosiospsis tetrandra* (Fig. 1c). *Milicia excelsa* (Fig. 1b) illustrates the restrictions of a limited field of view when the sample contains large and few vessel elements in the wood structure.

In all but one of the wood species studied, Inside Wood described simple perforations between vessel elements. The *Strombosiopsis tetrandra* sample was included in this study to provide additional information on the appearance of scalariform perforation plates. The pores distinctive for this type of perforation are very clear at the highest resolution and can still be counted at 3 µm. At 8 µm and 15 µm, the scalariform perforation can no longer be distinguished from a simple perforation. Fig. 1c shows a detail of the scalariform perforations in the *Strombosiopsis tetrandra* sample, both in the 3D rendering of the 1 µm scan and in the resliced planes at all four resolutions. Half of the studied species (9 out of 17) are described on Inside Wood as containing tyloses or deposits in the vessels. These could be observed at all four resolutions, as illustrated by the tyloses in *Vitex madiensis* (Fig. 2a)*.*

#### Pits

Pits between vessel elements, between vessels and rays or between fibre elements can be diagnostic features for wood identification. The small size of these pits (mostly smaller than 10 µm) means that they could generally only be seen at the highest resolution. Even at 1 µm however, the pits are not always distinguishable. A description of their distribution is possible in most samples at 1 µm, provided there are sufficient vessel elements in the scanned sample. Measuring the pits was only successful in half of the samples, depending on their size. Information about the appearance of their borders could not be inferred from the scans.

Minute pit size (described on the IAWA list of anatomical features as ≤ 4 µm) is a diagnostic feature for *Crossopteryx febrifuga*. Figure 1a illustrates the difference in appearance of these small pits between 1 µm and 3 µm resolution. The pits can be distinguished on the 1 µm scans, but not measured with enough precision to be used in the anatomical description. They are indistinguishable on the 3 µm scan. In contrast, Fig. 2b shows the scanned sample of *Ricinodendron heudelotii*. This species is described as having large pits (categorised on the IAWA list of anatomical features as ≥ 10 µm), which could indeed be measured at an average of 25 µm on the 1 µm scans and seen (but not measured) on the 3 µm scans.

#### Fibres

At the two higher resolutions, 1 µm and 3 µm, fibre elements can be distinguished, and the presence of septate fibres determined. At 8 µm and 15 µm, the individual fibres can no longer be distinguished. Determining the fibre wall thickness is also possible at 1 µm and 3 µm resolution, whereas it becomes more difficult at 8 µm and 15 µm. For example, the scan of *Strombosiopsis tetrandra* (Fig. 1c), shows thick fibre walls in the cross-sections of the higher resolutions, while this feature is less clear at the lower resolutions. The fibres of *Vitex madiensis* (Fig. 2a) are described on Inside Wood as thin-walled. The presence of septate fibres in this species can be observed at 1 µm and 3 µm, but no longer at 8 µm and 15 µm.

#### Axial parenchyma

All 17 scanned species included information on axial parenchyma in their Inside Wood descriptions. Apotracheal axial parenchyma cells, not associated with vessels, could be identified in all the samples with this feature at 1 µm, 3 µm and 8 µm, and in 70% of the samples at 15 µm resolution. Paratracheal axial parenchyma cells, surrounding the vessels, or banded axial parenchyma patterns could be observed in all the samples scanned at 3 µm and 8 µm. The small field of view offered by the 1 µm scans limited the information about the grouping of these cells, and features in these two categories could only be observed in half the samples with this description. At the lowest scanned resolution of 15 µm, the paratracheal axial parenchyma features could be described in more than half the samples presenting this feature. Of the banded parenchyma patterns, only the wide bands of 3 or more cells (IAWA feature code 85) could be observed at 15 µm. The strand length of the axial parenchyma cells could be clearly seen and counted at the highest resolutions, 1 µm and 3 µm. At 8 µm it was possible to count the strand length in 70% of the samples. At 15 µm, the parenchyma strands could no longer be viewed with enough precision to be counted.

Figure 2c shows the scans of a *Ficus mucuso* sample. The wide axial parenchyma bands are visible at all resolutions. The strand length can be counted on the tangential plane at 1 µm and 3 µm. Figure 3a shows the scans of *Nauclea latifolia,* with diffuse-in-aggregates axial parenchyma cells. This pattern can be recognized in the cross-sections at all resolutions. The number of cells in the axial parenchyma strands can be seen on the 1 µm and 3 µm scans.

#### Rays

On the tangential plane, the 1 µm and 3 µm scans allow to count the number of cells in a ray’s width clearly. At 8 µm, it becomes more difficult to distinguish these cells and at 15 µm the width of the rays can no longer be observed with any precision. Similarly, on the radial plane the composition of the ray cells could be described at 1 µm and 3 µm, whereas the procumbent and square cells became less defined at the two lower resolutions. Of the 17 species studied, 3 included ‘storied rays’. This feature could be observed in all three samples at 1 µm, 3 µm and 8 µm, but not at 15 µm. *Ficus mucuso* (Fig. 2c) is described as having larger rays (4 to 10 cells wide), and body ray cells procumbent with 2–4 rows of upright and/or square marginal cells. At all 4 resolutions the wide rays of the species can be observed, although at 15 µm the number of cells of the ray width can no longer be counted. Figure 3b shows a sample of *Pterocarpus angolensis*, presenting uniseriate rays. These can be observed at 1 µm and 3 µm but become less defined at 8 µm and disappear at 15 µm.

The quantitative features describing the rays, such as ray height and number of rays per mm^2^, could not be measured at the highest resolution, with a cross-sectional field of view of only 1 mm^2^, approximately. Some of the scanned species included features such as sheath cells, perforated ray cells and disjunctive ray parenchyma cell walls. Despite the advantage of the 3D nature of the scans, allowing a search through the scanned volume and increasing the probability of these, they weren’t observed in the scans at any resolution.

#### Inclusions

On the Inside Wood database, 10 of the 17 studied species were described as including secretory elements and cambial variants. Intercellular canals were not observed at any resolution. Laticifers or tanniniferous tubes, on the other hand, could be found in half the samples at 3 µm and 8 µm, yet more difficult to recognize at 1 µm and 15 µm. Fig. 2c shows this feature indicated with a blue circle on the 3 µm, 8 µm and 15 µm scans of the *Ficus mucuso* sample.

Inclusions such as crystals were hard to miss on the scans, ‘lighting’ up due the diffraction of X-rays. Even at the lowest resolution, the presence of crystals could be observed. The position of these crystals inside ray cells (procumbent, upright or both), axial parenchyma cells (chambered or non-chambered) or inside fibres, could be observed at 1 µm, 3 µm and 8 µm. Their shape could only be visualised in the 3D renderings at the highest resolution. Figure 3c shows the 3D rendering of the 1 µm scan of *Albizia zygia.* The prismatic crystals in the sample could be isolated from the wood elements and are indicated in blue. The position of the crystals in the axial parenchyma cells, as well as their shape, can be visualized in the 3D rendering, as well as in the radial and tangential slices at 1 µm, 3 µm and 8 µm. On the 15 µm scans, the presence of the crystals can be seen, but their location inside the rays is no longer discernible.

The presence of silica bodies could be discerned only at the highest resolution. Silica has a different attenuation than the surrounding wood structure but is not as bright as crystal elements and can’t be as clearly isolated in a rendering of the scan as was shown for crystals.

### Identification potential

Figure 4 shows that the resolution with the highest number of scans with ‘high identification potential’ (≤ 20 species returned by Inside Wood), is 3 µm voxel size. At 8 µm, the potential for a wood identification is also high, with only two scans resulting in more than 20 species. The 1 µm scans, although containing more detail, resulted in more than 20 species on the Inside Wood database for 5 of the 17 samples. The lowest potential for wood identification was for the lowest resolution, with 8 samples with more than 40 potential wood species. It must be noted, however, that even at the lowest resolution, for 6 samples the scans could be described with enough diagnostic features with < 20 species as a result.

## Discussion

### Comparing scan resolutions

The feature observation success rates combined with the Inside Wood exercise demonstrate that scans at 3 µm resolution have the highest potential for wood identification, with enough detail to distinguish small-scale features, but still a sufficient field of view to describe large-scale features. While the 1 µm scans offer the best view of small-scale features, their field of view is limited by the sample size restrictions to achieve this high resolution. The 15 µm scans do not contain enough diagnostic features to get a limited list of species from the Inside Wood database in more than half of the studied samples.

It is important to note that the results from the Inside Wood exercise are not fully representative of an identification of an unknown specimen. In such a case, the absence or presence of a feature must be interpreted, rather than confirmed. While, based on the Inside Wood exercise, scans between 3 µm and 8 µm are optimal for wood identification, in some cases small-scale features only visible at 1 µm resolution can be diagnostically important. When studying a sample small enough to scan at 1 µm, it is therefore preferable to acquire multiple scans at this resolution, or scan at two different resolutions, as this will offer both a high-resolution quality and a larger surface area to study. For most objects, however, the highest achievable resolution will be lower than the advised 3 µm to 8 µm in this paper. Therefore, when 3 µm scans are inconclusive and when subsampling is allowed, taking a small subsample is interesting. Although invasive, only a small subsample is needed to be scanned at the highest resolution. In addition, as this subsample will be unchanged by the scanning process, information from the scans can be supplemented with additional methods of analysis to get the most information on the wood species.

### Opportunities of 3D scans

The conventional microscopic study of wood anatomy offers a 2D snapshot of the wood structure, limited to the thin section cut from the sample. Yet X-ray µCT scanning results in three-dimensional information of the wood structure that can be digitally explored and oriented in any direction [15, 35].

This proved instrumental, for example, in the observation of features such as vessel and fibre elements. The scans enable us to follow these elements in the wood structure and digitally slice them at any angle. Thus, the scans allow the largest surface of connecting tissue between two vessels or a vessel and a ray to be found, revealing the best surface to view pits. Even at the highest resolution scanned in this experiment, however, the borders of the pits could not be visualised. A sub-micron resolution is required for this feature description [36, 37]. Similarly, the path of the fibre elements can be traced in the scanned volume, even if they aren’t oriented perfectly axially. Where microscopic analysis requires macerations of the wood, the scans allow the fibres to be measured along the entire element [35, 38].

For the observation of rays on the radial and tangential planes, the three-dimensional nature of the scans also proved a great advantage. By ‘leafing’ through the digital volume, the rays can be tracked throughout the wood. Compared to the stationary nature of microscopic sections, this allowed a more thorough search and understanding of the rays’ width and composition. Especially in those wood species with wide rays, the presence and combination of procumbent and upright ray cells on the radial plane could be observed throughout several rays to best determine which IAWA category they matched with.

A further advantage of studying a 3D volume of wood for the observation of anatomical features is that there is an increased chance to find even those features that are less frequently or distinctly present. In the scanned volume of a wood sample, a larger, three-dimensional volume can be searched through compared with microscopic thin sections, and the presence or absence of rare features can be more confidently stated.

A final advantage is the non-destructiveness of X-ray µCT analysis: it leaves the internal wood structure unchanged. The preparation of thin sections for microscopy involves cutting and chemically colouring the sample and can cause damage to the wood structure that is then studied [39]. This became apparent during the analysis of the *Vitex madiensis* sample, shown in Fig. 2a. Tyloses can be observed in every vessel element in the scanned volume. In comparison, the thin sections found in the RMCA’s xylarium of the same reference sample (sample number Tw2033 in the xylarium) show tyloses present in only one of the vessel elements; although this may in part be due to the variation between sample positions.

### X-ray µCT versus microscopy

X-ray µCT scans show anatomical features differently than optical microscopy. The features are shown in monochrome grey-scale results, according to their attenuation coefficients, whereas microscopical thin sections can offer additional chemical information on the presence of lignin and cellulose [40]. It’s important to be aware of this discrepancy in appearance between some anatomical features on the X-ray µCT scans and the micrograph material traditionally used as a reference for wood species.

The distinctiveness of the growth ring boundaries is difficult to define in certain tropical wood species [41]. Of the 17 scanned species only 4 have distinct boundaries. X-ray µCT is sensitive to density variations [42, 43], thus an observed difference in density can be misinterpreted as a distinct growth ring.

Scalariform perforation plates can be an important diagnostic feature. In 16 of the 17 studied species, the perforation plates were described as simple, and were (seemingly) easy to recognise on the scans at all resolutions. However, the only species with scalariform perforations, *Strombosiopsis tetrandra*, showed that from 8 µm upward the bars in the perforation plates could no longer be distinguished (shown in Fig. 1c). Indeed, the scalariform perforation looked simple at the two lower resolutions.

It was also noted that when describing rays on the tangential plane at a resolution of 8 µm or lower, smaller rays will no longer be well-defined. It is possible, at these lower resolutions, to confuse the larger cells of the parenchyma strands for uniseriate rays. Crystal inclusions are easy to spot on X-ray µCT scans, while sometimes harder to find on thin sections. This enables an observation of both their position in the wood structure, and their shape (prismatic or other shapes).

## Conclusions

This paper demonstrates the potential of X-ray µCT to visualize the anatomical features and identify a wide range of wood species. Although the best results were obtained with scans at 3 µm and 8 µm voxel size, even the lowest tested resolution of 15 µm showed potential for wood identification. Only the scans at the highest resolution, 1 µm voxel size, offered information on small scale features, such as pits or fibres.

If the dimensions of a wood specimen allow a high-resolution scan at 1 µm or higher, multiple scans of the sample are recommended. This way the scans will offer highly detailed information of a large field of view. For larger wood specimens or objects that preclude a resolution under 8 µm, it may be preferable to take a small subsample to scan at a higher resolution, to increase the probability of capturing valuable anatomical information on the scans and securing a wood species identification. This method also offers the opportunity to apply other (destructive) techniques to the sample after scanning if needed.

The 3D nature of X-ray µCT scan provides a unique advantage to wood analysis. In contrast to the stationary and limited view of a thin section, the digital volume can be resliced in any direction, and allows browsingthrough a stack of images, increasing the likelihood of finding certain uncommon features. The findings outlined in this multi-resolution overview, as well as the creation of a reference database of scanned wood species, will aid in the development of a standardized and systematic approach towards wood identification using X-ray µCT, as well as help in developing artificial intelligence systems for automated wood identification.

## Data Availability

The datasets supporting the conclusions of this article are available in the Zenodo repository:—the database containing all cultural objects identified in the collection of the Royal Museum for Central Africa, referenced in the methods section: 10.5281/zenodo.10671337—all scans discussed in this paper can be found here: 10.5281/zenodo.10640661.

## Abbreviations

µCT: Micron and sub-micron computed tomography
RMCA: Royal Museum for Central Africa
XS: Indicating the transverse plane of wood (following the conventions in the RMCA wood department)
RLS: Indicating the radial plane of wood
TLS: Indicating the tangential plane of wood
